# Short-Term Efficacy of the Artificial Intelligence HeartBot II in Increasing Awareness and Knowledge of Heart Attack in Women: Protocol for a Randomized Controlled Trial With a Waitlist Control

**DOI:** 10.2196/103597

**Published:** 2026-08-12

**Authors:** Yoshimi Fukuoka, Diane Dagyong Kim, Jingwen Zhang, Thomas J Hoffmann, Kenji Sagae

**Affiliations:** 1 Department of Physiological Nursing, School of Nursing, University of California, San Francisco, 521 Parnassus Ave, San Francisco, CA, 94143, United States, 1 415476-8419; 2 Department of Communication, University of California, Davis, Davis, CA, United States; 3 Department of Epidemiology & Biostatistics, and Institute for Human Genetics, University of California, San Francisco, San Francisco, CA, United States; 4 Department of Linguistics, University of California, Davis, Davis, CA, United States

**Keywords:** artificial intelligence, intelligent systems, chatbot, natural language processing, large language models, machine learning, heart disease, mobile apps, women, randomized controlled trial

## Abstract

**Background:**

Heart disease remains a leading cause of death for women in the United States. Despite this burden, awareness that heart disease is the leading cause of death among women declined from 65% in 2009 to 44% in 2019, with the largest declines observed among Hispanic, Black, and younger women. Thus, innovative, scalable, and cost-effective educational strategies are needed to improve women’s awareness of heart attack symptoms and appropriate care-seeking behaviors.

**Objective:**

This study aims to evaluate the short-term efficacy of the artificial intelligence (AI) HeartBot II, a chatbot-based educational intervention, in improving women’s awareness and knowledge of heart attack symptoms and care-seeking behavior compared with a waitlist control group.

**Methods:**

This randomized controlled clinical trial (RCT) with a waitlist control will enroll 200 women aged 25 or older, who will be randomized using a 1:1 allocation ratio. The intervention group will download the AI HeartBot II app and complete the 4 modules (including information on heart attack symptoms, risk factors, and calling 911) over 12 weeks. The waitlist control group will start receiving an identical intervention at 12 weeks. The primary outcomes will be change from baseline to 12 weeks in a 4-item heart attack response preparedness score, calculated as the mean of 4 self-reported items assessing confidence in recognizing signs and symptoms of a heart attack, distinguishing heart attack symptoms from other medical problems, calling 911 or an ambulance if a heart attack is suspected, and reaching an emergency room within 60 minutes of symptom onset. The primary analysis will estimate the intervention effect using constrained longitudinal data analysis implemented with linear mixed models, including fixed effects for time and time-by-treatment group interaction. Sensitivity analyses for the individual ordinal items will use ordinal logistic mixed-effects models.

**Results:**

We received approval from the University of California, San Francisco, Institutional Review Board (No. 25‐44825) on January 9, 2026, and this trial was registered on ClinicalTrials.gov (NCT07416734) on February 11, 2026, prior to enrollment of the first participant. Recruitment began in April 2026. As of manuscript submission, 86 participants were enrolled. Enrollment is expected to be completed by September 2026, and all follow-up assessments are anticipated to be completed by March 2027. Data analysis is expected to begin in spring 2027, with study results anticipated for publication later in 2027.

**Conclusions:**

To the best of our knowledge, this is the first RCT to rigorously evaluate the efficacy of the AI HeartBot II intervention. If effective, AI HeartBot II could provide a scalable, accessible, and cost-effective public health communication strategy to improve women’s awareness of heart attack symptoms and promote timely care-seeking behaviors in the United States.

## Introduction

Artificial intelligence (AI) chatbots are computer programs designed to simulate human conversations via text, speech, or both. Early rule-based AI chatbots relied on scripted dialogue flows. However, recent advancements in natural language processing (NLP) and large language models (LLMs) have significantly expanded the capabilities of AI chatbots, such as having more flexible and human-like naturalistic conversations as well as an understanding of more complex user input. Furthermore, AI chatbots can provide personalized, scalable, round-the-clock communication, making them attractive tools for health care and research. Given such advantages, AI chatbots have been rapidly developed and adapted to enhance patient education, counseling, clinical decision-making, and health management efficiency. Recent systematic reviews and meta-analyses on AI chatbots have shown potential to improve mental health [1-3] and other chronic illnesses [4] and to promote healthy lifestyles and self-care behaviors [5-10].

Despite these advancements, the use of AI chatbots, especially those powered by LLMs, in the area of cardiovascular health education remains underexplored. Heart disease remains the number one cause of death for women in the United States [11], with over 60 million women affected [12]. Public health campaigns, such as “Go Red for Women” [13] by the American Heart Association (AHA), have aimed to raise awareness over the past two decades. However, awareness that heart disease is the leading cause of death among women declined from 65% in 2009 to 44% in 2019 [14], with the steepest declines observed in Hispanic, Black, and younger women [15-17]. Therefore, an urgent need exists for a novel approach to increase awareness and knowledge of heart disease in women [18,19].

To address this gap, we conducted a series of studies to evaluate the feasibility, acceptability, and potential efficacy of an AI chatbot (hereafter referred to as “HeartBot I”) in increasing women’s awareness and knowledge of heart attack symptoms and appropriate care-seeking behavior. The results of those studies and HeartBot I design details were published elsewhere [18,19]. In brief, HeartBot I was feasible (ie, no withdrawal from HeartBot I conversation) and accepted by women, and its interactions were significantly associated with improvements in awareness and knowledge of all heart attack outcomes. However, since HeartBot I was a rule-based chatbot, its behavior and responses were limited to content authored specifically for HeartBot I conversations. To improve HeartBot I’s capacity, we incorporated all women’s heart attack and relevant questions collected in our previous studies and expanded its knowledge bank to enable more educational conversations. To achieve more human-like, natural, and personalized conversations, we implemented a new version of AI HeartBot (hereafter referred to as “HeartBot II”) powered by an LLM, specifically gemini-2.5-flash [20] through the Google Conversational Agents platform [21]. Combined with the high prevalence of smartphone ownership [22], an LLM-based HeartBot II could have significant advantages over traditional public health campaigns. This study aims to evaluate the efficacy of the HeartBot II program, as compared to the waitlist control, in increasing women’s awareness and knowledge of heart attacks. The waitlist control group will start interacting with HeartBot II after 12 weeks and will receive an SMS text message once a week from the research team for the initial 12 weeks for attention control. This protocol outlines the design and methodology of the upcoming randomized controlled trial (RCT) with a waitlist control to assess the efficacy of an LLM-based HeartBot II in this context.

## Methods

### Study Design

**Figure 1.**
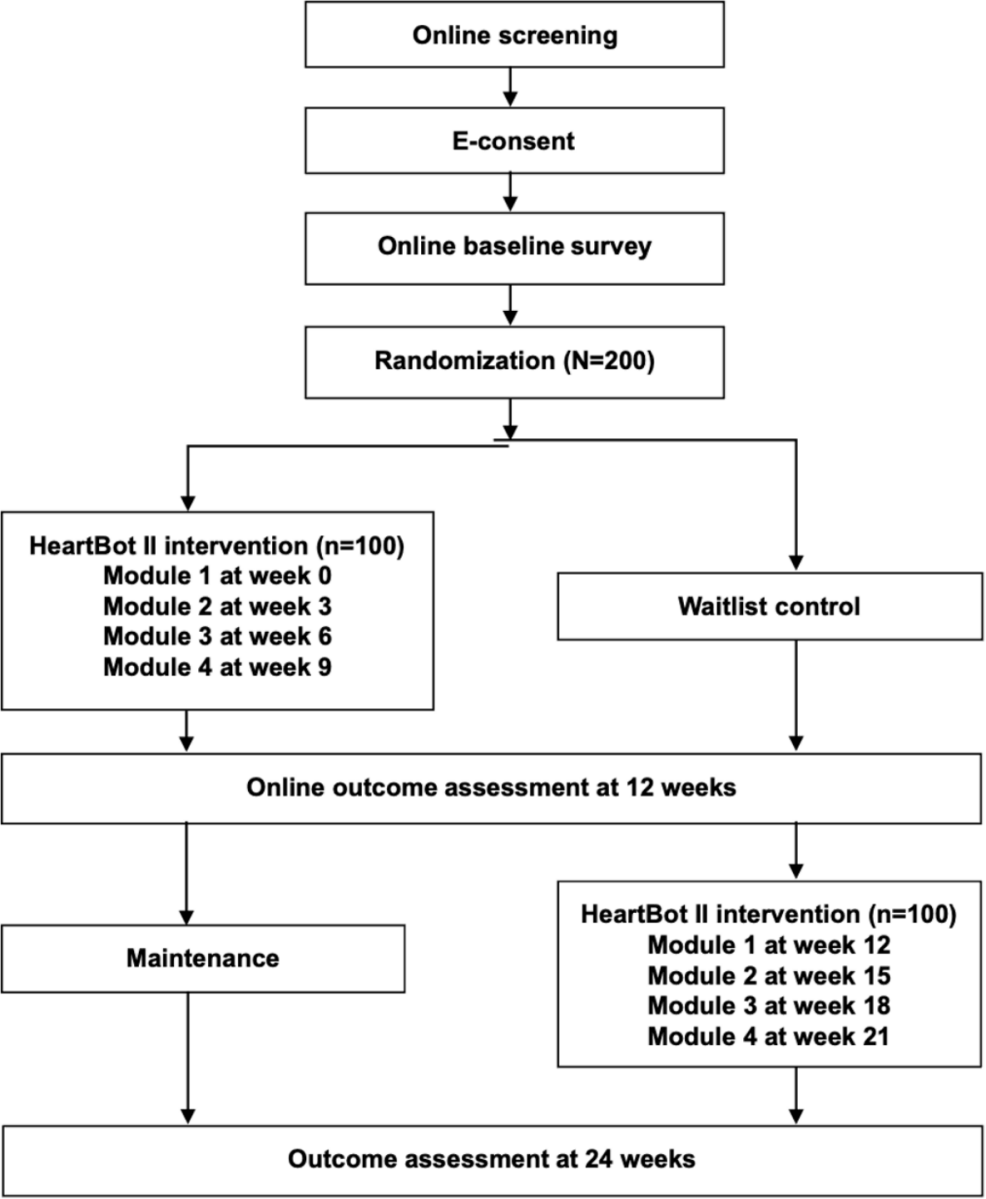
Study flow diagram and assessment schedule for the AI HeartBot II randomized controlled trial. AI: artificial intelligence.

An RCT with a waitlist control is being conducted to compare the efficacy of HeartBot II, as compared to the waitlist control, in increasing awareness and knowledge of heart attacks in women. Figure 1 shows the study design. This study was registered with ClinicalTrials.gov (NCT07416734) on February 11, 2026. All study procedures will be done online remotely.

### Ethical Considerations

This study was funded by the University of California, San Francisco (UCSF), Resource Allocation Program in February 2025. Correspondence from the peer review process associated with this funding award is provided in Peer Review Report 1. The study protocol was approved by the UCSF Institutional Review Board on January 9, 2026 (IRB number 25‐44825). This study was also registered with ClinicalTrials.gov (NCT07416734) on February 11, 2026. The SPIRIT (Standard Protocol Items: Recommendations for Interventional Trials) 2025 checklist of items to address in a randomized trial protocol is attached in Checklist 1.

### Eligibility Criteria for Participants

Participants will be eligible for inclusion if they meet all of the following criteria: (1) women aged 25 years or older; (2) no self-reported history of heart disease or stroke; (3) no terminal illness or diagnosed cognitive impairment, including Alzheimer disease; (4) not currently a health care professional or health care trainee; (5) not employed in the health care field; (6) residing in the United States and being a UCSF Health patient; and (7) possession of a smartphone with an active data plan or access to Wi-Fi.

### Recruitment and Setting

Recruitment will be conducted using the UCSF Clinical & Translational Science Institute (CTSI) recruitment services. Potential participants will be identified through the UCSF electronic medical record (EMR) and invited to participate via the MyChart messaging system. Individuals who open the invitation will be directed to a study information page and given the option to indicate interest in the proposed RCT. Interested individuals will be directed to an initial online eligibility screening survey administered through REDCap (Vanderbilt University).

### HeartBot I Development and Description

HeartBot I, the first version of HeartBot, was developed from collecting data through a Wizard of Oz experiment, in which a master-prepared cardiovascular nurse researcher acted the role of HeartBot and delivered the intervention contents to women through SMS text messages. After the Wizard of Oz experiment study and based on analyzing the conversational patterns and human inquiries, we designed and evaluated HeartBot I, a rule-guided, SMS-based conversational agent that delivers preauthored educational messages in a structured format [18,19]. In short, HeartBot I was designed with a language model component that was used to classify user utterances into intents and using a rule-based dialogue structure to deliver corresponding preauthored responses developed by the research team. We implemented it on the Google Dialogflow CX platform and linked to Twilio (Twilio Inc) for SMS text messaging conversation based on the intents and entities paradigm [23]. HeartBot I identified the general intent of each incoming message and responded with an appropriate, scripted reply. Although HeartBot I can recognize a range of user inputs, its responses are intentionally constrained to maintain accuracy and consistency in delivering heart disease education. We tested HeartBot I in 92 diverse women in a pre- and postintervention trial, and the findings of this study showed potential efficacy of HeartBot I [18,19].

### HeartBot II Development and Description Used in This Study

HeartBot II is an interactive, AI chatbot built on the Google Conversational Agents platform using an LLM, specifically gemini-2.5-flash, designed to simulate natural and human-like conversations. The system is designed to follow a script similar to that used in HeartBot I, but the use of an LLM allows it to be more natural and responsive in a wider range of conversational situations. The HeartBot II intervention will be delivered through a mobile app, allowing participants to engage with the chatbot at their convenience on their smartphones.

Table 1 provides an overview of the four modules of HeartBot II conversation contents. The HeartBot II intervention consists of four modules, to be completed sequentially over a 9-week period: module 1 (overview) at week 0, module 2 (women’s heart attack symptoms) at week 3, module 3 (risk factors) at week 6, and module 4 (prevention) at week 9. Each module takes approximately 10 to 15 minutes. Module 1 covers foundational education on heart attack, emphasizing the importance of early recognition and symptoms. Module 2 emphasizes the distinct symptoms that women often experience and the importance of a timely response. Module 3 addresses modifiable and nonmodifiable risk factors for heart disease. Module 4 focuses on prevention strategies such as physical activity and diet. Each module begins with a brief introduction and informs the user about what to expect in the conversation. For participant safety, the introduction message includes the following medical emergency notice at the start of each module: “If you are experiencing a medical emergency, please call 911 immediately.”

HeartBot II’s LLM foundation enables more flexible and adaptive conversations. Participants can type free-text responses, and HeartBot II generates replies that maintain personalization and relevance to the ongoing dialogue. The messages sent by HeartBot II (Table 1) are developed by cardiovascular experts based on the latest guidelines and evidence to ensure full control over the content presented to participants and to minimize the risk of the system dispensing false or misleading information. Although the system’s conversational behavior is managed by an LLM, the core content of what HeartBot II says is still drawn from materials authored by experts, including not just the canonical interaction script, but also a knowledge bank containing answers to common questions about heart disease. The knowledge bank currently includes 594 curated question-answer pairs, covering both study-related inquiries (eg, study procedures, data protection, participant expectations) and module-specific educational content related to heart disease.

In addition, we incorporate personalization and empathic responses, essential communication characteristics to improve participants’ experience and engagement [24]. Personalization features include the use of participants’ first name and dynamic responses based on previous user input. Empathetic communication is also embedded through conditionally triggered responses that acknowledge users’ personal experiences to simulate supportive and trustworthy interactions. For instance, when participants indicate that a close friend or family member has experienced a heart attack, HeartBot II generates supportive acknowledgments (eg, “*I’m sorry to hear that, [participant’s name]*”). To ensure readability, the content sent by HeartBot II (Table 1) has been evaluated using Flesch-Kincaid readability metrics. This analysis yielded a Flesch Reading Ease score of 64.3 and a Flesch-Kincaid Grade Level of 6.9 for module 1, 69.6 and 6.5 for module 2, 63.2 and 7.4 for module 3, and 61.1 and 7.7 for module 4, indicating that the language used for all four modules was accessible and comprehensible to a broad audience.

**Table 1.** Overview of AI HeartBot II educational content by module and message sequence.

Message order	Module 1: overview	Module 2: women’s heart attack symptoms	Module 3: risk factors	Module 4: prevention
1	Introduction and greetings	Introduction and greetings^+^	Introduction and greetings ^++^	Introduction and greetings ^+++^
2	Participants’ name retrieval	Participants’ name retrieval^+^	Participants’ name retrieval^++^	Participants’ name retrieval^+++^
3	Knowledge of heart attacks	Leading cause of death for women in the United States^+^	Risk factors for heart disease^+^	Presence or absence of tobacco and e-cigarette use
4	Symptoms of heart attacks	The most common symptom of a heart attack for women	Increased risk of heart disease	Presence or absence of regular physical activity
5	Leading cause of death for women in the United States	Other common symptoms of a heart attack in women	Female-specific risk factors for heart disease^+^	Perceived own weight category
6	Gender factors for heart attacks	First action when experiencing symptoms of a heart attack^+^	Racial and ethnic differences in women’s heart disease risk^+^	Body Mass Index self-check
7	First action when experiencing symptoms of a heart attack	Importance of calling 911^+^	Heart disease screening tests	Presence or absence of diabetes^++^
8	Importance of calling 911	Time to seek medical help^+^	Presence or absence of high blood pressure	Latest total cholesterol level
9	Time to seek medical help	Presence or absence of chest pain during heart attack	Cholesterol test	Symptoms and signs of high blood pressure
10	Treatment of heart attacks	Dizziness, nausea, and shortness of breath as heart attack symptoms	Presence or absence of diabetes	Awareness to Dietary Approaches to Stop Hypertension (DASH) diet
11	Action plans while waiting for 911	Indigestion as heart attack symptom in women	Family history of heart attack	Selection of modifiable heart disease risks
12	Risk factors for heart disease	Calling 911 before contacting others	Presence or absence of depression or depressive symptoms	Intention to planning to visit health care provider
13	Female-specific risk factors for heart disease	Recognizing heart attack	Intention to reduce own heart attack risks	Multiple choice questions^+++^
14	Racial and ethnic differences in women’s heart disease risk	Communicating heart attack symptoms in the emergency department	Plan for cholesterol screening	Further questions to ask HeartBot II^+++^
15	Multiple choice questions	Talking to a male doctor in the emergency department	Further questions to ask HeartBot II^++^	Acknowledgment and conclusion of the conversation^+++^
16	Further questions to ask HeartBot II	Multiple choice questions^+^	Acknowledgment and conclusion of the conversation^++^	—
17	Acknowledgment and conclusion of the conversation	Further questions to ask HeartBot II*	—	—
18	—^a^	Acknowledgment and conclusion of the conversation*	—	—

a not available.

### HeartBot II Safety Considerations

Since HeartBot II is a conversational app involving an LLM, special consideration is given to safety issues regarding user data and the information provided by the system. Unlike general-purpose open-domain AI chatbots, HeartBot II operates within a narrow domain, which allows us to constrain the chatbot’s behavior to minimize risks commonly associated with AI chatbots. Because HeartBot II’s conversational engine is built on the Google Conversational Agents framework, both the research team’s content and all user input, including any content provided to or generated by LLMs in our system, is protected by Google Cloud’s enterprise data governance and privacy policies. Importantly, HeartBot II content and user input are kept private and are never used to train foundation models. Additionally, HeartBot II makes full use of the safety guardrails provided in the Conversational Agents platform, including: detection and filtering of common responsible AI harm categories (hate speech, dangerous content, sexually explicit content, and harassment), which are set to the highest and most strict settings; and protection against jailbreak and prompt injection attempts [25,26].

Unlike many conversational AI apps that rely primarily on an AI model’s knowledge and developer prompts, HeartBot II relies crucially on a knowledge bank that comprises the system’s domain knowledge. To prevent hallucination or dispensing of information not reviewed by the research team, HeartBot II is designed to provide users only with information included explicitly in its instructions, which include a detailed interaction script, and its knowledge bank, which allows the system to answer questions that fall outside the interaction script. The Conversational Agent’s platform provides a grounding setting that allows system developers to set a level of confidence that information provided by the system comes from a given data store. In HeartBot II, the strictest settings are used to ensure content comes from its knowledge bank [27]. Finally, as a final and definitive safeguard, a member of the research team will review all sessions in their entirety at least twice a week. In case incorrect or otherwise problematic information is given to a user, a member of the research team will contact the user by SMS text message and email to alert the user and provide a correction.

### Intervention Versus Waitlist Control

#### Waitlist Control Group

The waitlist control group will not receive the HeartBot II intervention during the initial 12-week period, but will receive an SMS text message once a week for 12 weeks for attention control. These text SMS messages do not include any educational content related to heart attack or heart health and are limited to neutral study reminders and general self-report prompts. For example, at week 3, participants will receive the following message: “Hi! How would you rate your overall health on a scale from 1 to 8? 1 means very poor, and 8 means excellent. Please reply with one number.” In week 9, participants will receive the following message: “Hi! You’ve reached Week 9 of the study. Thank you for staying involved. You will download the study app at Week 12 and use it four times. For now, please text us your biggest current health concern (for example, high blood pressure or weight gain).” Following completion of the week 12 outcome assessments, participants in the waitlist control group will initiate the HeartBot II program, delivered identically to the intervention group.

#### Intervention Group

Participants assigned to the intervention group will receive the HeartBot II intervention during the first 12 weeks. The intervention group will download the app on their smartphone and will be asked to start Module 1 immediately after the randomization. The detailed content and timing of the intervention are described in the section below.

#### Mobile HeartBot II App

The HeartBot II intervention will be delivered through a mobile app available for both iOS and Android operating systems. The iOS version will be distributed via the Apple app store, and the Android version will be distributed via the Google Play Store. Participants randomized to the intervention group will be provided with direct download links to the appropriate app store as part of the intervention onboarding process.

**Figure 2.**
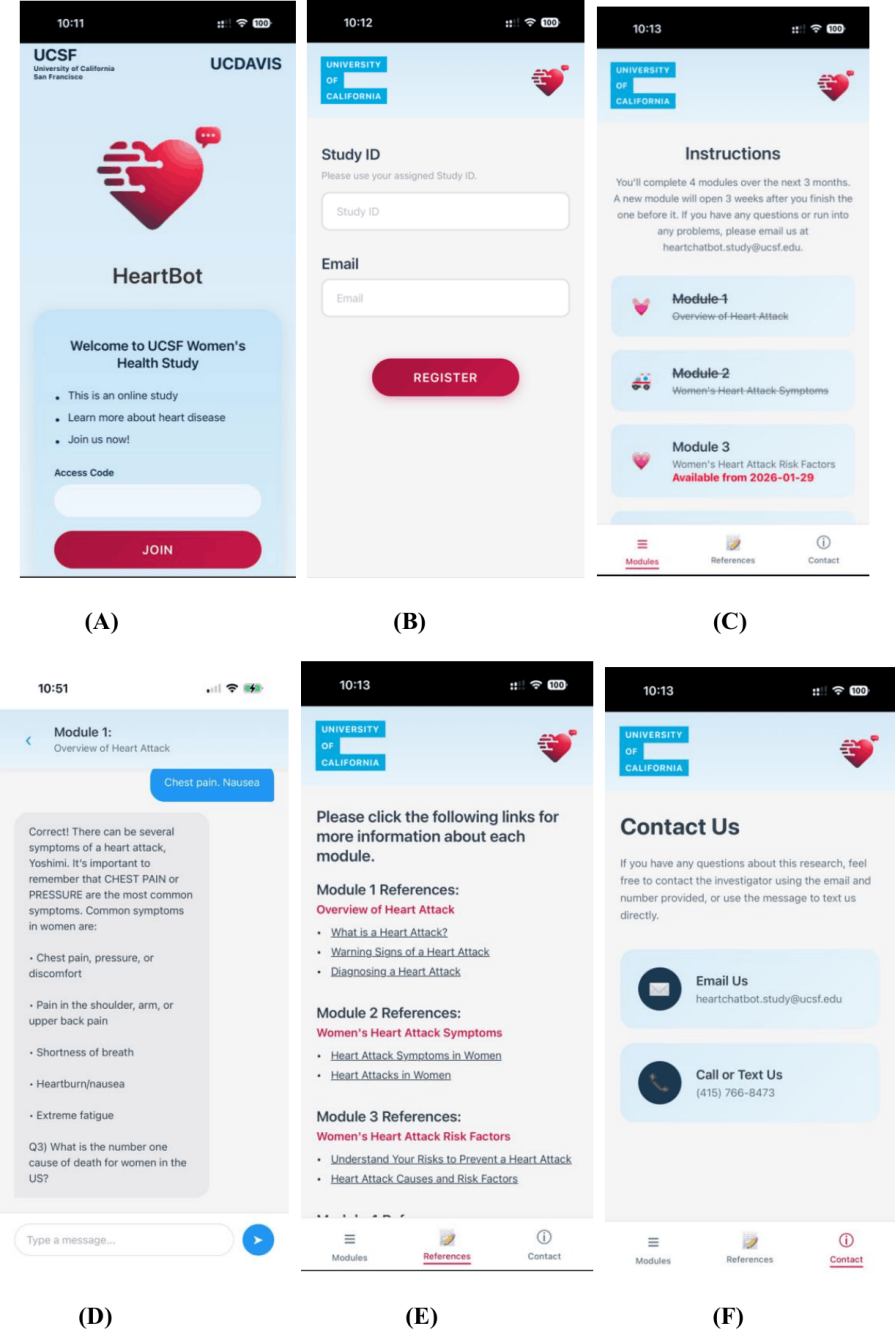
Representative screenshots of the AI HeartBot II mobile app. AI: artificial intelligence; UCSF: University of California, San Francisco.

Figure 2 presents screenshots of the HeartBot II app interface. After downloading the app, participants will be required to enter a unique access code to activate the app (Figure 2A). This access code will be generated by the research team and is provided only to individuals who have successfully completed the eligibility screening, consent form, and baseline survey. The access code will serve as an authentication mechanism to restrict app access to enrolled participants and to ensure that intervention exposure is limited to study participants.

Upon entering the access code, participants proceed to the registration screen, where they will be asked to enter their assigned study identification number and email address. These identifiers will be used solely for study management purposes and allow the research team to securely link participants’ app-based interactions and module completion data with survey data collected through REDCap. No personally identifiable information beyond the study email is displayed within the app.

Following successful registration, participants are directed to the app home screen, which provides an overview of the study procedures and displays the 4 HeartBot II educational modules (Figure 2C). Each module contains a chatbot focused on a specific topic related to women’s heart health. Modules are unlocked sequentially according to the study timeline, and participants can clearly see which modules are available, upcoming, or completed. To promote timely module completion and improve adherence, participants will receive automated notifications when a new HeartBot II module becomes available. Notifications will be delivered via both app push notifications and email.

Selecting a module opens a dedicated chat interface (Figure 2D), in which participants engage in a structured conversation with HeartBot II. The interface is designed to resemble commonly used mobile messaging apps to enhance familiarity and usability. Messages generated by HeartBot II appear in gray message bubbles, while participant responses appear in blue message bubbles. Participants interact with the chatbot by typing free-text responses.

In addition to the chatbot conversation, the app includes supplemental features to support participant learning and communication with the research team. The “References” tab (Figure 2E) provides links to credible educational resources related to each module topic. The “Contact Us” tab displays email and phone contact information of the research team, which allows participants to seek assistance with technical issues or study-related questions throughout the intervention period (Figure 2F).

### Procedure

#### Online Screening and E-Consent

All potential participants who are interested in the study will complete an online screening form to determine eligibility based on the study’s inclusion and exclusion criteria. The screening will be reviewed by research staff. Participants who meet all eligibility criteria will be asked to sign the e-consent form. Individuals who do not meet eligibility criteria or who do not respond to the invitation will not proceed further in the study.

#### Baseline

After obtaining the signed consent form, the trained research staff will ask participants to complete a baseline online, REDCap, survey. The survey will assess sociodemographic characteristics, baseline knowledge and awareness of heart disease and symptoms, health behaviors, and other relevant behavioral variables. Survey responses will be used to evaluate within- and between-group changes over time; upon completion of the baseline survey, participants will be randomly assigned to either the intervention or waitlist control group.

#### Randomization

200 women will be randomly assigned in a 1:1 ratio to the HeartBot II intervention or a waitlist control group. Permuted-blocked randomization will be used to ensure balance between groups, with block sizes varying randomly among 2, 4, and 6. The randomization scheme will be generated and implemented in REDCap by a staff member who is not involved in participant recruitment, intervention delivery, data collection, or outcome assessment. Investigators and research staff will be blinded to block size and allocation sequence. The individual preparing the randomization scheme has no role in evaluating or executing the experiment. Participants will be informed of their assignment after randomization.

#### Blinding

Because of the behavioral nature of the intervention, participants cannot be blinded to group assignment. However, they will not be informed of the specific study hypotheses or primary outcome measures. The randomization sequence will be generated and implemented in REDCap by a staff member not involved in recruitment, intervention delivery, or outcome assessment. Investigators and research staff will remain blinded to allocation sequence and block sizes. Outcome data are self-reported via REDCap.

#### Data Management and Data Monitoring

All survey data will be collected and managed using REDCap, a secure, web-based application hosted by UCSF. Data entry will occur directly by participants through REDCap, minimizing transcription errors. Built-in range checks (eg, age upper and lower limits), logic checks, and required response settings will be implemented to enhance data quality. Data completeness will be reviewed regularly by research staff. Heartbot II conversational data and app usage data will be securely stored on a University of California, Davis-managed Amazon Web Services (AWS) server approved for research use. Access is restricted to authorized users and applications using certificate-based credentials, and all communications are encrypted to maintain confidentiality and integrity. Conversational data, AWS-stored app data, and REDCap survey data are stored separately within their respective secure databases. Datasets are linked only through unique anonymous participant identification numbers, minimizing the risk of participant re-identification. Access to all study data is limited to authorized study personnel who have completed the required human subjects protection and data security training. All data will be stored on secure, password-protected servers in accordance with institutional data security policies.

#### First 12-Week Period

Participants in the HeartBot II intervention group will begin the HeartBot II module 1 immediately following randomization. The intervention will be delivered over 9 weeks and will include four modules, with each module scheduled approximately 3 weeks apart. Participants will receive reminders at the beginning of each module and will be encouraged to complete each conversational session at their own pace within the designated timeframe. In addition to the guardrails placed on the system through the Conversational Agents platform and automatic review of session transcripts to ensure health information in the system’s responses come directly from the carefully controlled content in the system’s knowledge bank, research staff will closely monitor the participants’ responses during the HeartBot II conversations at least twice a week to ensure that hallucination does not occur to compromise participants’ safety. The waitlist control group will not receive any intervention during this period, but will receive monthly reminders informing them that they will begin the HeartBot II program after 12 weeks. At the end of the 12-week period, all participants will be invited to complete the 12-week online survey for the outcome assessment. This survey will measure primary and secondary outcomes.

#### 12- to 24-Week Period

Following the completion of the 12-week online survey, participants in the intervention group will enter a maintenance phase and will not receive further chatbot interactions. In addition, they will receive weekly SMS text message reminders reinforcing key heart attack knowledge and prompting completion of the final online survey at week 24. The waitlist control group will begin the HeartBot II intervention, following the same 4 modules schedule as the intervention group starting at week 12. They will receive the same instructions and reminders throughout the 12- to 24-week period. At week 24, all participants will complete the final online survey. For the intervention group, this represents a follow-up assessment three months postintervention; for the waitlist control group, it represents the follow-up assessment 3 weeks postintervention. If all participants complete all study requirements, they will receive a US $20 Amazon e-gift card. To minimize the burden on participants’ time, all study procedures will be done online remotely. Data collected during the surveys will be captured on the REDCap system.

#### Primary Outcome Measures

The primary outcome will be change from baseline to 12 weeks in a 4-item heart attack response preparedness score, calculated as the mean of four items assessing confidence in (1) recognizing heart attack symptoms, (2) distinguishing heart attack symptoms from other medical problems, (3) calling 911 or an ambulance if a heart attack is suspected, and (4) reaching an emergency department within 60 minutes of onset. The measure has been adapted from a previously validated scale [28,29], which has been used in prior studies with diverse samples of women to support generalizability [30-32]. Participants will answer the following four questions on a 4-point scale, ranging from 1, in which 1 indicates “not sure,” to 4, which indicates “sure”: (1) How sure are you that you could recognize the signs and symptoms of a heart attack in yourself? (2) How sure are you that you could tell the difference between the signs or symptoms of a heart attack and other medical problems? (3) How sure are you that you could call an ambulance or dial 911 if you thought you were having a heart attack? (4) How sure are you that you could get to an emergency room within 60 minutes after the onset of your symptoms? All items are self-reported and collected online. In pilot data [18], the 4-item scale demonstrated acceptable internal consistency (Cronbach α=0.70).

#### Key Secondary Outcome Measures

Key secondary outcomes will be the change from baseline to 12 weeks in each of the four individual items comprising the 4-item heart attack response preparedness score. These item-level analyses will be conducted as prespecified key secondary endpoints to characterize which components of the summary score are most affected by the intervention.

#### Secondary Outcome Measures

To evaluate user perceptions of the HeartBot II intervention, we will include several validated and study-developed measures that assess chatbot communication quality, message perception, and user attitudes toward AI in health.

#### HeartBot II Message Effectiveness

Message effectiveness will be assessed using the effectiveness scale [33,34], which consists of five semantic differential items (effective—ineffective, helpful—unhelpful, beneficial—not beneficial, adequate—not adequate, supportive—not supportive). Each item will be rated on a 7-point scale, with 1 indicating the negative pole and 7 indicating the positive pole. Scores will be averaged to create a composite score, with higher values indicating greater perceived message effectiveness.

#### Impression of Chatbot Messages

Perceived humanness of chatbot messages will be measured using the anthropomorphism scale [35], consisting of five bipolar adjective pairs: (fake—natural, machinelike—humanlike, unconscious—conscious, artificial—lifelike, rigid—adaptive). Items will be rated on a 7-point scale, and scores will be averaged to compute a composite humanness score, where higher scores reflect more mechanical impressions of the chatbot.

#### Conversational Naturalness and Coherence

Two single-item measures will assess perceived conversational quality: “Overall, how would you rate the conversations with your texting partner?” (1=Very unnatural to 5=Very natural), “Overall, how would you rate the messages you received?” (1=Very incoherent to 5=Very coherent).

#### Chatbot Identity Perception

Participants will be asked to identify who they believe they interacted with during the intervention: “Do you think you texted a human or artificial intelligent chatbot during your conversation?*”* with two response options: (1) human and (2) AI chatbot.

#### AI Attitudes in Health Context

To explore perceptions of AI in health care, participants will be asked to rate agreement with the following three statements using a 5-point Likert scale (1=Strongly disagree, 5=Strongly agree): “The use of artificial intelligence will result in better healthcare,”“The use of artificial intelligence will result in better health outcomes,” “Artificial intelligence may help me reduce my risk of heart disease.”

### Other Measures

#### Perceived Risk of Heart Attack

Perceived susceptibility will be assessed using three items asking participants to compare their risk of experiencing a heart attack to other women their age over different time frames (next 5 y, next 10 y, lifetime). Responses will be measured on a 5-point Likert scale from “Very unlikely” to “Very likely.”

#### Sociodemographic and Health Risk Factors

Basic demographic information, including age, race and ethnicity, education, income, marital status, and employment status will be self-reported. Cardiovascular risk factors will also be assessed, including smoking status, current use of prescribed blood pressure, cholesterol, or diabetes medication, and family history of heart disease. These items were selected based on current clinical guidelines [36].

#### Multiplicity and Gatekeeping Procedure

To control the family-wise error rate, a hierarchical gatekeeping procedure will be used for the primary and key secondary outcomes. First, the group-by-time interaction for the heart attack response preparedness score will be tested at a two-sided alpha level of 0.05. Only if the primary outcome is statistically significant will the four individual component items be formally tested as key secondary outcomes. *P* values for the four key secondary outcomes will be adjusted using the Holm-Bonferroni step-down procedure. All other secondary outcomes will be treated as exploratory; analyses will be descriptive or inferential, as appropriate, with nominal p-values reported without adjustment for multiplicity.

#### Statistical Analysis

Baseline characteristics will be summarized by randomized group using counts and percentages for categorical variables and means and standard deviations for continuous variables, or medians and interquartile ranges for skewed variables. Because treatment assignment is randomized, baseline characteristics will be described primarily to assess sample comparability rather than to guide significance-based inference.

Primary efficacy analyses will compare randomized groups from baseline to 12 weeks, before the waitlist group receives the intervention. The treatment effect for the primary outcome, a 4-item heart attack response preparedness score, will be estimated using constrained longitudinal data analysis (cLDA) implemented via linear mixed models (LMMs) [37]. Consistent with the randomized design, the models will constrain the mean scores for the treatment and control groups to be equal at baseline. The primary outcome will include categorical fixed effects for time and a time-by-treatment group interaction term. The primary model contrast of interest is the coefficient for this interaction term at the 12-week follow-up, representing the baseline-adjusted between-group difference. To account for the within-subject correlation between baseline and follow-up, the cLDA models will use an unstructured covariance matrix, allowing variances and correlations to differ over time.

The 4 individual preparedness items will be analyzed as prespecified key secondary outcomes, subject to the hierarchical gatekeeping procedure described above. The primary efficacy analysis and all key secondary analyses will be based on the baseline and 12-week assessments only. Although the individual items are ordinal, the primary analysis will use the same linear cLDA framework to facilitate interpretation of mean differences and support model stability [38]. Robustness of this linear approximation will be evaluated in sensitivity analysis using ordinal logistic mixed-effects models for each individual item.

All analyses will follow the intent-to-treat (ITT) principle, with all randomized participants in the groups to which they were assigned. Missing data will be addressed using multiple imputation by chained equations (MICE) to preserve the repeated-measures structure of the data. Multiple imputation was selected in part to address occasional missing item-level responses when constructing the composite preparedness score. Imputation will be conducted in a wide format, using predictive mean matching (PMM) so that the imputed values for the 4-point Likert items remain restricted to observed response categories. The imputation model will include treatment assignment, timepoint-specific observed item values, baseline scores, and related outcome items as predictors. The primary preparedness score will be calculated within each imputed dataset only after item-level imputation. We will generate 50 imputed datasets to reduce Monte Carlo error, and results will be combined across imputations using Rubin’s rules.

To evaluate how app engagement and module completion influence efficacy, the main ITT analyses will be supplemented by an exploratory per-protocol analysis. App engagement will be operationalized as binary module completion (completing all 4 modules vs fewer than 4 modules). In the per-protocol analysis, an unconstrained linear mixed model analogous to the primary model will be fit, restricting the intervention group to “high engagers” (participants who completed all 4 educational modules), and comparing them with the full waitlist control group. An exploratory dose-response analysis will also be conducted by examining the association between the number of completed modules (0 to 4) and the 12-week preparedness score within the intervention arm using an unconstrained linear mixed model analogous to the primary analysis. Because intervention adherence is not randomized, these analyses will be considered exploratory; the cLDA constraint of equal baseline means will not be imposed, and results will be interpreted cautiously.

Exploratory analysis of the 24-week assessment will evaluate the maintenance effects among participants originally randomized to HeartBot II and within-group changes after they receive the intervention. Because the waitlist control group receives the intervention after week 12, 24-week between-group comparisons also lack randomized control protection. Therefore, exploratory analysis of all available repeated measurements (baseline, 12 wk, and 24 wk) will be conducted using an unconstrained linear mixed model with a participant-specific random intercept and fixed effects for treatment group, timepoint, and their interaction. Because treatment exposure is no longer randomized after week 12, these analyses will be considered exploratory and descriptive rather than confirmatory.

Exploratory secondary outcomes will be analyzed using methods appropriate to their scale and distribution. Continuous secondary outcomes will be analyzed using linear regression or linear mixed models, as appropriate to the timing of measurement. Binary or categorical secondary outcomes will be analyzed using generalized linear models or generalized linear mixed models.

#### Power and Sample Size

This study is powered based on a total analyzed sample of n=160 (80 per group). To account for a conservative 20% attrition, we will enroll a total of 200 participants. Statistical power was estimated via Monte Carlo simulations (5000 iterations) using an empirical covariance matrix and response thresholds derived from pilot data [18]; code is provided in Multimedia Appendix 1. The simulation uses the same cLDA analysis framework described above for analysis.

For the primary outcome, the heart attack response preparedness score, the study provides 90% power to detect a raw mean difference of 0.37 points on the 1‐4 scale (Cohen *d*=0.51). This detectable effect is smaller than the within-group mean change observed in pilot data (0.78 points), although the pilot study did not include a control group [18]. For the four key secondary item-level outcomes, power was also evaluated under the prescribed hierarchical gatekeeping procedure with Holm-Bonferroni adjustment. Under this framework, the study has 90% power to detect at least one significant key secondary item for a raw difference of 0.43 (*d*=0.59). To detect significant effects across all four key secondary items (assumed to have the same effect), larger effect sizes are required; 90% power is achieved at a mean difference of 0.62 points (*d*=0.85).

## Results

Participant screening and enrollment began in April 2026. As of June 2, 2026, recruitment is ongoing, and 86 participants have been enrolled. Enrollment is expected to be completed by September 2026, and all follow-up assessments are anticipated to be completed by March 2027. Data analysis is expected to begin in spring 2027, with study results anticipated for publication later in 2027.

## Discussion

### Anticipated Findings

The proposed RCT will evaluate the efficacy of AI HeartBot II, a large language model–based chatbot intervention designed to increase women’s awareness and knowledge of heart attack symptoms and appropriate care-seeking behaviors. We hypothesize that participants assigned to the HeartBot II intervention will demonstrate greater improvements in heart attack response preparedness, symptom recognition, and confidence in seeking timely emergency care than participants assigned to the waitlist control group. We also anticipate that participants will report favorable perceptions of the chatbot’s usefulness, communication quality, and overall acceptability.

Heart disease remains the leading cause of death among women in the United States. However, awareness of cardiovascular disease risk and heart attack symptoms continues to decline, particularly among younger women and women from racial and ethnic minority groups. If effective, HeartBot II may represent a scalable, accessible, and cost-effective approach for delivering evidence-based cardiovascular health education to large populations of women through smartphones.

### Comparison With Prior Work

Previous studies have demonstrated the potential of AI chatbots to improve health knowledge, promote healthy behaviors, and support chronic disease self-management. Systematic reviews have reported promising effects of conversational agents across a variety of health domains, including mental health, physical activity, smoking cessation, and chronic disease management [1-10]. However, relatively little research has focused on AI-driven interventions for cardiovascular health education, particularly among women.

Most published studies involving cardiovascular applications of LLMs have evaluated the accuracy and quality of AI-generated responses to clinical or patient questions from prevention to treatment. Although these studies suggest substantial promise, concerns remain regarding misinformation, hallucinations, and inconsistent responses. Consequently, there is a need for rigorously designed intervention studies that evaluate not only the technical performance of AI systems but also their effectiveness in improving patient-centered outcomes. HeartBot II builds upon our previous work evaluating HeartBot I, a rule-based chatbot that demonstrated feasibility, acceptability, and preliminary efficacy in improving women’s awareness and knowledge of heart attacks [18,19]. The present trial extends this line of research by incorporating a LLM to support more natural, personalized, and engaging interactions while maintaining expert-developed educational content.

### Strengths and Limitations

This study has several strengths. First, the RCT design provides a rigorous framework for evaluating intervention efficacy. Second, the intervention is grounded in prior formative research and builds upon an earlier version of HeartBot that demonstrated promising preliminary outcomes. Third, the mobile app format allows broad accessibility and scalability while supporting personalized and conversational interactions. Fourth, the intervention content was developed by cardiovascular experts and is based on current evidence and clinical guidelines, thereby reducing the risk of misinformation.

Despite these strengths, several limitations should be considered. First, while HeartBot II leverages LLMs to enable more naturalistic conversations, its educational messages are delivered through four predesigned, structured modules created by the research team to ensure safety, quality, and intervention fidelity. As such, HeartBot II may not fully accommodate user-initiated inquiries that diverge from the scope of the modules. For example, users seeking highly personalized information, such as individual risk assessments or advice tailored to their medical histories, may find the chatbot less responsive. Although the chatbot’s conversation management is powered by an LLM, allowing for flexible and natural interaction, the system is designed to dispense only information authored by expert content authors and found explicitly in the system’s knowledge bank. Second, the primary outcomes rely on self-reported measures of awareness, knowledge, and preparedness rather than objective behavioral or clinical outcomes. The study population is recruited from a single health system and includes women with smartphone access, which may limit generalizability. Finally, the intervention is currently delivered through a text-based interface, and future versions may benefit from multimodal features such as voice interaction, audiovisual content, or adaptive personalization.

### Future Directions

If HeartBot II demonstrates efficacy, future research should examine its effectiveness in more diverse populations and health care settings, including community-based and underserved populations. Additional studies should evaluate longer-term retention of knowledge, effects on actual care-seeking behavior, and potential impacts on cardiovascular risk reduction. Future iterations of HeartBot II may also incorporate multimodal communication capabilities, individualized risk assessment tools, and integration with electronic health records or clinical workflows to enhance personalization and reach.

### Dissemination Plan

Study findings will be disseminated through peer-reviewed publications, presentations at national and international scientific conferences, and reports to relevant stakeholders. Results may also inform future collaborations with health systems, public health organizations, and community partners interested in leveraging AI-based interventions to improve cardiovascular health education among women.

### Conclusions

This protocol describes a RCT evaluating the efficacy of AI HeartBot II, a large language model–based chatbot designed to improve women’s awareness and knowledge of heart attack symptoms and appropriate emergency response behaviors. By combining evidence-based cardiovascular education with conversational AI technology, HeartBot II has the potential to provide a scalable, accessible, and cost-effective approach to public health communication and surveillance. The findings from this trial will contribute important evidence regarding the role of AI-powered chatbots in cardiovascular health promotion and may inform the future development and implementation of digital interventions aimed at reducing disparities in women’s cardiovascular health.

## Supplementary material

10.2196/103597 Multimedia Appendix 1 Power simulation code for the statistical analysis section.

10.2196/103597 Checklist 1 SPIRIT 2025 checklist of items to address in a randomized trial protocol.

10.2196/103597 Peer Review Report 1 Peer review report by the UCSF Resource Allocation Program.

## Abbreviations

AHA: American Heart Association
AI: artificial intelligence
AWS: Amazon Web Services
cLDA: constrained longitudinal data analysis
ITT: intent-to-treat
LLM: large language model
NLP: natural language processing
RCT: randomized controlled trial
REDCap: Research Electronic Data Capture
SPIRIT: Standard Protocol Items: Recommendations for Interventional Trials
UCSF: University of California, San Francisco
