# Supplementary material for: Short-Term Efficacy of the Artificial Intelligence HeartBot II in Increasing Awareness and Knowledge of Heart Attack in Women: Protocol for a Randomized Controlled Trial With a Waitlist Control
Source: JMIR Res Protoc. 2026 Aug 12;15:e103597. doi: 10.2196/103597 (PMC13465624; doi:10.2196/103597)
Supplement: Supplementary file 1 [file resprot-v15-e103597-s003.pdf]

Review Committee

Digital Health Research

Final Score

1.3

Summary of Discussion

Comments from **Reviewer A**

1. General critique and summary of the proposal.

This is a new application for a Pilot for Established Investigators by Dr. Yoshimi Fukuoka. This application requests $50,000 for a 2-year pilot study examining feasibility and preliminary effect of an AI chatbot ("HeartBot"). The HeartBot is an autonomous chatbot delivered via SMS text message and is available 24/7. The intervention takes place as a text message conversation between the participant and the chatbot to explore existing knowledge about heart disease and deliver educational materials dynamically based on personal beliefs and gaps in knowledge. This proposal is a pilot RCT that plans to enroll 160 diverse women (75% from minoritized ethnic/racial groups) – 80 to the chatbot intervention arm and 80 to the waitlist control arm. Participants in the intervention group will be asked to complete the first conversation with the HeartBot within 48 hours after completing the baseline survey, then again every 3 weeks. The HeartBot is programmed to cover specific topics related to heart disease education (e.g., what is a heart attack, heart attack symptoms) and each message was crafted manually to ensure full control over the content delivered. The waitlist control arm will also receive the HeartBot intervention after 3 months. Primary outcomes of knowledge and self-efficacy of recognizing symptoms of heart attack and heart disease will be assessed with validated AHA questionnaires. Minor weaknesses of this application include lack of clarity in the analytic plan, use of a waitlist control group (as opposed to a more active control group), and lack of targeted recruitment for younger women; however, these only modestly dampen the high enthusiasm for this application.

2. Significance. Does the study address an important problem?

Yes, the applicant outlined recent research showing that awareness of heart disease as the leading cause of death in women in the U.S. has drastically declined in recent years. There are also important disparities in this decline in knowledge and awareness such that the lowest levels of heart disease awareness are found in Black/African American, Hispanic, and young (25- to 34-year-old) women. These women are also less likely to recognize their heart attack symptoms and more likely to die from their first heart attack than men. This proposal importantly addresses these disparities head on by focusing the study on a diverse group of women, with targeted goals to recruit 160 women with 75% representation of those from racially/ethnically minoritized groups.

3. Approach. Are methods, study design and analysis appropriate?

The application is based on an “AI Chatbot Behavior Change Model” that the applicant and their team previously published, which incorporated feedback from women to guide the design of a chat-bot based behavioral change program for heart disease knowledge and awareness. The current proposed RCT is the next step in the process to evaluate this “HeartBot” for feasibility and preliminary effectiveness in a diverse sample of women with cardiovascular risk. They will enroll 160 women from diverse groups who will be randomized to the AI HeartBot intervention or waitlist control group. The SMS approach is strong, as it is low-cost and does not require smartphone ownership. Prelimary data support the need for these targeted approaches among young and racially minoritized women, their ability to recruit mostly women from minoritized groups, preliminary feasibility of the HeartBot with high rates of trial completion and reported acceptance, and significant increases in heart attack awareness. The HeartBot is designed in such a way that puts conversational control in the hands of the system, not the user/participant to ensure that conversations are predictable and is more control over what the chatbot can do and say (unlike generative AI chatbots like ChatGPT) – this is also a strength of the study. Dropout/missingness is handled appropriately in the analytic plan; however, the analytic plan lacks some clarity and details about the planned linear mixed models. For example, it is unclear whether the applicant will specifically test for differences in the change in outcomes by group (intervention vs. control) and how. Relatedly, this was unclear because the study design did not specify whether the waitlist control group would also have 2 timepoints of outcomes assessments at the start and end of their waitlist period. Other minor weaknesses include: 1) the use of a waitlist control group as opposed to a stronger active control group (e.g., participants who would still interact with some other chatbot or perhaps receive different educational materials), though this is understandable given the scope of this pilot RCT; and 2) the lack of targeted recruitment for younger women despite the disparities among young women *in addition* those from minoritized racial/ethnic groups.

4. Innovation. Is the study original and innovative?

Yes. This will be among the first studies to evaluate a chatbot-based intervention to improve awareness and knowledge of heart disease in women. This chatbot uses natural language processing to deliver the intervention via text message, which is a low cost and very scalable approach with potential to have large reach even among harder-to-reach, more vulnerable groups. In addition, the applicant states that although this intervention is currently English-based, the program could be easily adapted to other languages.

5. Investigator(s). Is the applicant(s) trained to do the studies? Does the applicant have a strong track record in research?

The PI, Dr. Fukuoka, conducts a research program focused on primary prevention of cardiovascular disease in women using AI. She has a strong, longstanding track record of funding from AHRQ, NINR, NHLBI, NIDDK, National Science Foundation, American Heart Association, and others. Other research team members include co-investigators Dr. Jingwen Zhang, Dr. Kenji Sagae, and Dr. Holli DeVon, who have complementary interdisciplinary expertise in communications, chatbot development, computational linguistics, and sex differences of acute coronary syndromes. All members of the research team have a strong track record in research and obtaining extramural funding. There are no weaknesses noted in this team of investigators.

6. Environment/Departmental support. Is appropriate support available from department? Does the applicant(s) have access to the necessary tools?

The departmental letter of support is strong. Dr. Fukuoka and team have the available resources to successfully carry out this study. No weaknesses noted.

7. Future potential. Is the research likely to lead to extramural funding?

This study has excellent potential to lead to extramural funding because of both the innovation and scalability of the AI HeartBot intervention as well as the success and motivation from the research team. Dr. Fukuoka has already discussed future funding opportunities with program officers from the Patient-Centered Outcome Research Institute and the Agency for Healthcare Research and Quality.

8. Any question or concern about the budget?

None noted.

Comments from **Reviewer B**

---

1. General critique and summary of the proposal.

While heart disease is the leading cause of death among women, awareness that this is the case among women has been declining over time. This project aims to increase awareness and understanding of cardiovascular risk and heart disease among women using an AI chatbot. Presumably, the increased awareness of heart disease among women will indirectly be associated with decreased heart disease related mortality and morbidity among women. The investigators will perform a randomized control study comparing knowledge gain associated with use of their chatbot to assess for feasibility with detailed plans for expansion to extramural funding.

This is an exceptionally strong study that is well-considered with an innovative intervention. The study team brings relevant experience and expertise.

2. Significance. Does the study address an important problem?

Yes. The authors have identified declines in awareness and knowledge about heart disease risk among women and have targeted their intervention to address that in a personalized way.

3. Approach. Are methods, study design and analysis appropriate?

The study is well-considered, with an intervention group and a control group although both groups get the intervention eventually. The participants will be block randomized and the sample size seems sufficiently large for a pilot. While no power calculation is given, power is arguably not possible without first conducting this study. There is a plan for recruitment in place and pre-intervention assessment. The study will use a modified version of a validated scale to assess heart disease risk knowledge. They also have an excellent analysis plan in place. One area with potential for improvement would be making the control group a true control group rather than a waitlist control group. From a results standpoint, the second knowledge assessment could be even stronger without a waitlist group.

4. Innovation. Is the study original and innovative?

Yes. The group systematically created and piloted a closed chatbot that seems ready for testing within this study.

5. Investigator(s). Is the applicant(s) trained to do the studies? Does the applicant have a strong track record in research?

Yes. Extensive experience in the topic and has already done relevant pilot groundwork.

6. Environment/Departmental support. Is appropriate support available from department? Does the applicant(s) have access to the necessary tools?

There are several relevant letters of support that describe extensive working relationships among the investigators.

7. Future potential. Is the research likely to lead to extramural funding?

Yes

8. Any question or concern about the budget?

No

© 2026 The Regents of the University of California
