# Supplementary material for: Short-Term Efficacy of the Artificial Intelligence HeartBot II in Increasing Awareness and Knowledge of Heart Attack in Women: Protocol for a Randomized Controlled Trial With a Waitlist Control
Source: JMIR Res Protoc. 2026 Aug 12;15:e103597. doi: 10.2196/103597 (PMC13465624; doi:10.2196/103597)
Supplement: Supplementary file 2 [file resprot-v15-e103597-s001.docx]

Supplementary code: Power simulations

```
library(MASS)
library(glmmTMB)
library(future.apply)
plan(multisession)

# Correlation matrix from pilot data
cor_data <- c(
  1.000, 0.683,  0.260, 0.281, 0.248, 0.291, 0.095,  0.194, # item1 pre
  0.683, 1.000,  0.306, 0.286, 0.224, 0.317, -0.004, 0.119, # item2 pre
  0.260, 0.306,  1.000, 0.499, 0.130, 0.127, 0.259,  0.278, # item3 pre
  0.281, 0.286,  0.499, 1.000, 0.028, 0.036, 0.047,  0.277, # item4 pre
  0.248, 0.224,  0.130, 0.028, 1.000, 0.641, 0.240,  0.259, # item1 post
  0.291, 0.317,  0.127, 0.036, 0.641, 1.000, 0.220,  0.191, # item2 post
  0.095, -0.004, 0.259, 0.047, 0.240, 0.220, 1.000,  0.532, # item3 post
  0.194, 0.119,  0.278, 0.277, 0.259, 0.191, 0.532,  1.000  # item4 post
)

rho_mat <- matrix(cor_data, nrow = 8, ncol = 8, byrow = TRUE)

# Parameters
breaks     <- c(-Inf, -0.785, 0.078, 0.888, Inf)  # From pilot data
n_per_grp  <- 80    # N after attrition
reps       <- 5000
target_diff <- 0.37
batch::parseCommandArgs()

set.seed(42)

# cLDA wrapper using glmmTMB
# makes assumptions on time and grp as simulated further below
run_clda_fast <- function(val, time, grp, id) {
  fit <- try(glmmTMB(
    val ~ time + time:grp + us(0 + time | id), # var at each timepoint
    dispformula = ~ 0, # standard companion for us(...)
    data = data.frame(val, time, grp, id)), silent = TRUE)

  if (inherits(fit, "try-error"))
    return(NA)

  # Extract p-value for the interaction term (TimePost:Group1)
  coefs <- summary(fit)$coefficients$cond
  target_row <- grep("^timepost:grp1$|^grp1:timepost$", rownames(coefs))

  return(coefs[target_row, "Pr(>|z|)"])
}

# Convert each column of latent conts item responses into observed
# ordinal item responses using specified cutpoints.
# Output is matrix of integer category scores (1-4 here) on observed scale.
latent_to_ord <- function(latent_mat, breaks) {
  apply(latent_mat, 2, function(x) as.numeric(cut(x, breaks = breaks)))
}


# Calibrate a latent shift to the observed composite difference.
# Then after discretizing to 1-4 ordinal scale, and averaging,
# the treatment-control difference on the observed score scale
# is equal to the target_diff
calibrate_latent_shift <- function(target_diff, rho_mat, breaks, n_big = 200000) {
  # large synthetic dataset to estimate from
  latent_big <- mvrnorm(n_big * 2, mu = rep(0, 8), Sigma = rho_mat)

  # how far resultsing observed post-treatment composite difference
  # is from the target difference
  objective_fn <- function(delta) {
    latent_tmp <- latent_big

    # second half of rows = treatment group, push by delta
    latent_tmp[(n_big + 1):(2 * n_big), 5:8] <-
      latent_tmp[(n_big + 1):(2 * n_big), 5:8] + delta

    # convert latent to observed
    ord_tmp <- latent_to_ord(latent_tmp, breaks)

    # compute composite difference
    comp_control_post <- rowMeans(ord_tmp[1:n_big, 5:8, drop = FALSE])
    comp_treat_post   <- rowMeans(ord_tmp[(n_big + 1):(2 * n_big), 5:8, drop = FALSE])

    # return difference of observed mean difference and targetted difference
    mean(comp_treat_post) - mean(comp_control_post) - target_diff
  }

  # solve for the latent shift delta
  uniroot(objective_fn, interval = c(0, 3), tol = 1e-4)$root
}

# Get the latent shift from above function
latent_push <- calibrate_latent_shift(
  target_diff = target_diff,
  rho_mat = rho_mat,
  breaks = breaks
)

# run the simulation
results <- future_replicate(reps, {
  # generate latent data
  latent <- mvrnorm(n_per_grp * 2, mu = rep(0, 8), Sigma = rho_mat)

  latent[(n_per_grp + 1):(n_per_grp * 2), 5:8] <-
    latent[(n_per_grp + 1):(n_per_grp * 2), 5:8] + latent_push

  # discretize
  ord_data <- apply(latent, 2, function(x) as.numeric(cut(x, breaks = breaks)))

  # reshape for cLDA
  ids  <- factor(rep(1:(n_per_grp * 2), each = 2))
  grp  <- factor(rep(c(0, 1), each = n_per_grp * 2))
  time <- factor(rep(c("pre", "post"), n_per_grp * 2), levels = c("pre", "post"))

  # primary outcome
  comp_pre <- rowMeans(ord_data[, 1:4])
  comp_post <- rowMeans(ord_data[, 5:8])
  val_comp <- as.vector(rbind(comp_pre, comp_post))
  p_primary <- run_clda_fast(val_comp, time, grp, ids)

  # four key secondaries (individual items)
  p_secondaries <- sapply(1:4, function(i) {
    val_item <- as.vector(rbind(ord_data[, i], ord_data[, i+4]))
    run_clda_fast(val_item, time, grp, ids)
  })

  p_secondaries[is.na(p_secondaries)] <- 1  # safety, failed fits are not significant

  # primary
  primary_sig <- !is.na(p_primary) && p_primary < 0.05

  # secondaries
  if (primary_sig) {
    p_adj <- p.adjust(p_secondaries, method = "holm")
    n_sig <- sum(p_adj < 0.05)
  } else {
    n_sig <- 0
  }

  comp_sd <- sd(comp_pre)

  return(c(
    primary_sig = as.numeric(primary_sig),
    sec_atleast1 = as.numeric(n_sig >= 1),
    sec_atleast2 = as.numeric(n_sig >= 2),
    sec_atleast3 = as.numeric(n_sig >= 3),
    sec_all4 = as.numeric(n_sig == 4),
    comp_sd = comp_sd))
}, future.seed = TRUE)

# output summary
power_summary <- rowMeans(results[1:5, ], na.rm = TRUE)
avg_comp_sd <- mean(results["comp_sd", ])
names(power_summary) <- c("Primary_Comp", "Sec_AtLeast1", "Sec_AtLeast2", "Sec_AtLeast3", "Sec_All4")

print(floor(power_summary * 100))

cat("Target composite mean difference:", target_diff, "\n")
cat("Average baseline composite SD:", round(avg_comp_sd, 3), "\n")
cat("Approximate Cohen's d:", round(target_diff / avg_comp_sd, 3), "\n")
cat("Calibrated latent shift:", round(latent_push, 3), "\n")
```
